# ERCP-related perforations: a population-based study of incidence, mortality, and risk factors

**DOI:** 10.1007/s00464-019-06966-w

**Published:** 2019-09-26

**Authors:** Ann Langerth, Bengt Isaksson, Britt-Marie Karlson, Jozef Urdzik, Stefan Linder

**Affiliations:** 1 Department of Surgical Sciences, Uppsala University, 751 85 Uppsala, Sweden; 2 Division of Surgery, CLINTEC, Karolinska Institutet, Center for Digestive Diseases, Karolinska University Hospital, Stockholm, Sweden

**Keywords:** ERCP, Adverse event, Perforation

## Abstract

**Background:**

Perforations related to endoscopic retrograde cholangiopancreatography (ERCP) are rare but feared adverse events with highly reported morbidity and mortality rates. The aim was to evaluate the incidence and outcome of ERCP-related perforations and to identify risk factors for death due to perforations in a population-based study.

**Methods:**

Between May 2005 and December 2013, a total of 52,140 ERCPs were registered in GallRiks, a Swedish nationwide, population-based registry. A total of 376 (0.72%) were registered as perforations or extravasation of contrast during ERCP or as perforation in the 30-day follow-up. The patients with perforation were divided into fatal and non-fatal groups and analyzed for mortality risk factors. The case volume of centers and endoscopists were divided into the upper quartile (Q4) and the lower three quartile (Q1–3) groups. Furthermore, fatal group patients’ records were reviewed.

**Results:**

Death within 90 days after ERCP-related perforations or at the index hospitalization occurred in 20% (75 out of 376) for all perforations and 0.1% (75 out of 52,140) for all ERCPs. The independent risk factors for death after perforation were malignancy (OR 11.2, 95% CI 5.8–21.6), age over 80 years (OR 3.8, 95% CI 2.0–7.4), and sphincterotomy in the pancreatic duct (OR 2.8, 95% CI 1.1–7.5). In Q4 centers, the mortality was similar with or without pancreatic duct sphincterotomy (14% vs. 13%, *p* = 1.0), but in Q1–3 centers mortality was higher (45% vs. 21%, *p* = 0.024).

**Conclusions:**

ERCP-related perforations are severe adverse events with low incidence (0.7%) and high mortality rate up to 20%. Malignancy, age over 80 years, and sphincterotomy in the pancreatic duct increase the risk to die after a perforation. The risk of a fatal outcome in perforations after pancreatic duct sphincterotomy was reduced when occurred at a Q4-center. In the case of a complicated perforation a transfer to a Q4-center may be considered.

Endoscopic retrograde cholangiopancreatography (ERCP) is a common procedure for treating diseases of the biliary and pancreatic ducts. The use of therapeutic ERCP has increased 30-fold in recent decades [1]. Together with per-oral cholangiopancreatoscopy, ERCP is also used to investigate complex pancreatobiliary diseases such as bile duct strictures and intraductal papillary mucinous neoplasms of the pancreas [2]. This increase in procedure complexity raises the requisite level of expertise needed to complete the procedure successfully and probably increases the risk of adverse events. Thus, understanding the factors associated with failed ERCP interventions is of great interest.

The success rate of ERCP varies markedly between institutions [3] and one study showed that lower endoscopist volumes were associated with higher failure rate for ERCP, and a greater need for post-procedure hospitalizations [4]. A systematic review and meta-analysis showed an association between endoscopist volume and adverse event rates including perforations [5]. However, there are no recommendations or guidelines concerning the specific minimum endoscopist or center volumes needed to maintain ERCP competences.

The short-term ERCP complication rate is reported to be around 10% [6] and includes pancreatitis, cholangitis, bleeding, and perforation. ERCP also carries an overall mortality rate between 0.1 and 6% [7–11]. Long-term complications have not been well investigated but acute pancreatitis and cholangitis seem to increase after endoscopic sphincterotomy. A possible increased risk for malignancy in the bile duct, papilla, and pancreas has also been discussed.

ERCP-related perforations are uncommon severe adverse events with an incidence between 0.1 and 1.5% [12–16]. In previous studies, factors, such as sphincterotomy, long duration of the procedure, high age, sphincter of Oddi dysfunction, and pre-cut, were reported to increase the risk for ERCP-related perforations [17, 18].

Death after ERCP-related perforations is most frequently reported to be around 10% [19–22], but mortality as high as 36% has been presented [23]. Late recognition of a perforation and the failure to adequately treat a perforation seem to worsen the outcome, especially in periampullary lesions [24].

The aim was to evaluate the incidence and outcome of ERCP-related perforations and to identify risk factors for death due to perforations in a population-based study.

## Materials and methods

Between May 2005 and December 2013, 52,140 ERCPs were registered in the Swedish, population-based registry for gallstone surgery and ERCP (GallRiks). The registry was established in 2005 and uses an internet platform (GallRiks, www.ucr.uu.se/gallriks), with online data registration of the procedures and 30-day follow-up information. Since the beginning of the registry the validity of the data is monitored by independent reviewers who visit the participating hospitals at least once every third year. The data registered here represent more than 90% of all ERCPs performed in Sweden [7]. Background data were extracted from GallRiks concerning all (52,140) registered ERCPs during the study period (Table 1).

**Table 1:** Patient and procedure characteristics for all ERCPs performed during study period

	All ERCPs
	*n* = 52,140
Age, mean ± SD, years	67 ± 17
Sex, *n* (%)	
Female	28,070 (54)
ERCP indication, *n* (%)	
Malignancy diagnosis	4957 (10)
Stent dysfunction	2632 (5)
Cholangitis	4192 (8)
Obstructive jaundice	11,151 (21)
Bile duct stone	16,828 (32)
Postoperative bile leakage	1404 (3)
Papilla within diverticula, *n* (%)	5543 (11)
Bile duct stenosis, *n* (%)	10,729(21)
Sphincterotomy, *n* (%)	
Bile duct	27,971 (54)
Pre-cut	4166 (8)
Pancreatic duct	1044 (2)
Bile duct stone extraction, *n* (%)	16,130 (31)
Procedural time, mean ± SD, min	36 ± 25

**Fig. 1:**
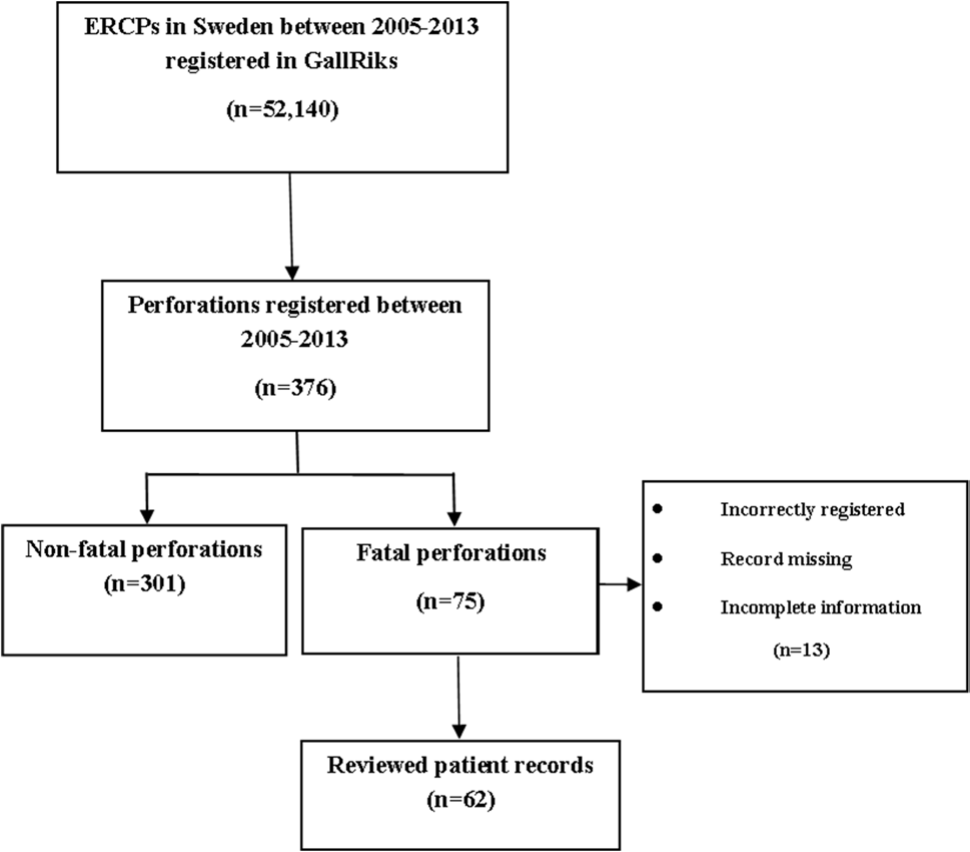
Flowchart concerning all included ERCPs and perforations

Perforations and contrast extravasations during the ERCP or as perforations during 30-day follow-up were registered (Fig. 1). All patients could then be identified by their national registration number, unique for each resident in Sweden [25]. Linkage to the validated [26] Inpatient Registry, containing information on all in-hospital treatment in Sweden, was made. Data were retrieved from the Cause of Death Register, also run by the Swedish National Board of Health and Welfare. According to the ethical approval, permission was only obtained to retrieve patient records for the fatal perforations.

The patients who died within 90 days after perforation or at the index hospitalization were identified and defined as the fatal group. The fatal group was then compared with patients surviving their perforations, i.e., non-fatal perforations. Additional information concerning malignancy was obtained from the Inpatient Registry due to possible overlapping diagnosis regarding the indication for ERCP, i.e., the same patient can have both jaundice and malignancy as an indication.

In the fatal group, the patient records were reviewed providing additional information, such as medication, comorbidity, and treatment after perforation. Five patient records were missing; five patients had incomplete information in the patient records, and three patients were incorrectly registered. In order to validate the fatal perforation group, the data of the 62 patients with confirmed perforations were compared to the 13 patients with missing or incorrect data (Fig. 1).

In order to stratify the center and endoscopist volumes, appropriate data were retrieved from GallRiks registry for the year 2013, when the registry had the most completed data during the study period. Based on the volumes, distribution, centers, and endoscopists were divided into groups within upper quartile (Q4) and the lower three quartiles (Q1–3). Four centers performed between 380 and 780 ERCPs corresponding to a quarter of all 7971 ERCPs during year 2013; other 47 centers performed between 10 and 320 ERCPs during the same period (Q1–3). At endoscopist level, ERCP volume during the study period varied between 2 and 696 for the first three quartiles Q1–3 of distribution and 721 up to 1487 ERCPs in the upper quartile Q4, which roughly corresponds to up to 83 ERCPs per year and endoscopist in Q1–3 and between 84 and 171 ERCPs per year and endoscopist in Q4.

Injuries were stratified according to the standardized Stapfer classification system (Table 2), as it is the most common classification system of duodenal perforation after ERCP. Complications that could not be classified according to Stapfer were named unclassified. GallRiks’ definitions of complications were used. Management after a perforation was divided into early, late, or best supportive care/no treatment. Early treatment was defined as treatment performed at the same time as the injury occurred, i.e., directly connected to the ERCP. All other surgical, endoscopic, or drainage treatment was defined as late treatment.

**Table 2:** Classification of iatrogenic perforations during ERCP

Type	Description
Stapfer	
I	Lateral or medial duodenal wall perforations, caused by the endoscope
II	Periampullary perforations
III	Distal bile duct injuries related to instrumentation
IV	Retroperitoneal air alone
Unclassified	Includes perforations of the esophagus, gastric perforations, stent perforation of duodenum, and the results of duodenal perforations other than those described above

The study was approved by the Regional Ethics Committee in Uppsala, Sweden (Protocol No. 2014/096/2).

### Statistics

The patients’ clinical data were expressed as mean ± standard deviation (SD) for the whole population, but as a median and interquartile range for study cohort or as a number and percentage. Mann–Whitney test was used for continuous parametric data. Categorical data were compared using the *χ*^2^ test or Fisher’s exact test when appropriate. Univariate and multivariate logistic regression analyses were used to investigate potential risk factors to die in perforation. Analyses were performed using the SPSS version 24.0 for Windows (SPSS Inc., Chicago, IL). A two-tailed *p* value of < 0.05 was considered statistically significant in all analyses.

## Results

A total of 376 cases (0.72%) were registered as perforations or contrast extravasations during the ERCP or as perforations during 30-day follow-up (Fig. 1). Death within 90 days after ERCP-related perforations or at the index hospitalization was 20% (75 out of 376) for perforations and 0.14% (75 out of 52,140) for all ERCPs. Thus, there were 301 non-fatal perforations.

Patient and procedure data for the fatal and the non-fatal perforations were compared and they are presented in Table 3. The fatal perforation group was significantly older with malignancy as the most common indication. Gender distribution was similar in both groups. Cholangitis and malignancy were the most common indications in the non-fatal group. Bile duct strictures were more common in the fatal group compared with the non-fatal group, but the frequency of stricture dilations was similar.

**Table 3:** Patient, procedure, and post-procedure characteristics for fatal compared to non-fatal perforations

	All perforations	Fatal perforations	Non-fatal perforations	*p* value
	*n* = 376	*n* = 75	*n* = 301	
Age, median (Q1; Q3), years	69 (56; 80)	77 (71; 84)	66 (52; 78)	< 0.001
Sex, *n* (%)				0.243
Male	158 (42)	36 (48)	122 (40)	
Female	218 (58)	39 (52)	179 (60)	
ERCP indication, *n* (%)				< 0.001
Malignancy diagnosis^a^	123 (33)	55 (73)	78 (26)	
Stent dysfunction	38 (10)	6 (8)	32 (11)	
Cholangitis	98 (26)	8 (11)	90 (30)	
Obstructive jaundice	17 (4)	6 (8)	11 (4)	
Bile duct stone	71 (19)	7 (9)	64 (21)	
Postoperative bile leakage	21 (6)	0	21 (7)	
Papilla within diverticula, *n* (%)	44 (12)	8 (11)	36 (12)	0.615
Bile duct stenosis, *n* (%)	131 (35)	37 (49)	94 (31)	0.012
Dilation biliary stricture, *n* (%)	41 (11)	6 (8)	35 (12)	0.367
Biliary stent, *n* (%)	134 (36)	28 (37)	106 (35)	0.732
Sphincterotomy, *n* (%)				
Bile duct	207 (55)	31 (41)	176 (58)	0.009
Pre-cut	86 (23)	21 (28)	65 (22)	0.282
Pancreatic duct	34 (9)	11 (15)	23 (8)	0.071
Bile duct stone extraction, *n* (%)	67 (18)	5 (7)	62 (21)	0.004
Procedural time, median (Q1; Q3), min	45 (30; 68)	45 (27; 66)	46 (30; 69)	0.296
Performing center, *n* (%)				
Q1–3		59 (23)	197 (77)	
Q4		16 (13)	104 (87)	0.028
Performing endoscopist, *n* (%)				
Q1–3		62 (22)	214 (78)	
Q4		13 (13)	87 (87)	0.042
In-hospital care after ERCP				
Days, median (Q1; Q3)	22 (10; 42)	21 (12; 33)	22 (9; 47)	0.125
Occasions, median (Q1; Q3)	3 (1; 4)	1 (1; 2)	3 (2; 5)	< 0.001

*Q* quartile

^a^Additional information acquired from the inpatient registry

In the fatal perforation group, sphincterotomy in the bile duct and bile duct stone extraction rates were lower. There was no difference in pre-cut frequency between the two groups. Sphincterotomy in the pancreatic duct showed trend to be more frequent in the fatal than the non-fatal perforation group. Number of occasions and duration of hospital care are presented in Table 3. Bleeding and pancreatitis were the most common additional adverse events. In the fatal group the frequencies were similar, 8 (11%) and 6 (8%) cases, respectively. In the non-fatal group, pancreatitis was more frequent than bleeding, with 54 (18%) and 16 (5%) cases, respectively. Forty-two of the 75 fatal perforations (56%) were recognized during ERCP and in the non-fatal group 182 (60%) (*p* = 0.802).

Risk factors to die in a perforation in the multivariate analyses were malignancy diagnosis, age > 80 years, and sphincterotomy in the pancreatic duct (Table 4).

**Table 4:** Univariate and multivariate logistic regression analysis comparing fatal perforations with non-fatal perforations

	Univariate logistic regression			Multivariate logistic regression		
	OR	CI 95%	*p* value	OR	CI 95%	*p* value
Age > 80 years	2.558	1.498–4.369	0.001	3.842	1.999–7.386	< 0.0001
Age	2.558	1.498–4.369	0.001			
Sex						
Male						
Female	1.354	0.815–2.251	0.242			
ERCP indication						
Malignancy diagnosis	7.827	4.413–13.883	< 0.001	11.203	5.819–21.569	< 0.0001
Papilla within diverticula	0.704	0.306–1.617	0.408			
Bile duct stenosis	2.396	1.417–4.053	0.001			
Dilation biliary stricture	0.661	0.267–1.635	0.370			
Biliary stent	1.096	0.649–1.851	0.732			
Sphincterotomy						
Bile duct	0.500	0.299–0836	0.008			
Pre-cut	1.412	0.795–2.507	0.239			
Pancreatic duct	2.077	0.964–4.479	0.062	2.839	1.078–7.478	0.035
Bile duct stone extraction	0.275	0.107–0.711	0.008			
Performing center						
Q1–3						
Q4	0.514	0.282–0.937	0.030			
Performing endoscopist						
Q1–3						
Q4	0.516	0.270–0.986	0.045			

*Q* quartile, *OR* odds ratio, *CI* confidence interval

Pancreatic sphincterotomy perforations were significantly more frequent among Q4 endoscopists than among the Q1–3 endoscopists but did not differ depending on center volume (Table 5). Q4 centers had no differences in mortality if a pancreatic duct sphincterotomy was performed or not, unlike Q1–3 centers which had a higher mortality after pancreatic duct sphincterotomy. Sphincterotomy in the pancreatic duct was performed in 34 cases and 11 of these developed post-ERCP pancreatitis; however, there were no significant differences concerning fatal outcome or not (*p* = 0.304).

**Table 5:** Perforation after sphincterotomy in the pancreatic duct according to center and endoscopist volume

	Total	No pancreatic sphincterotomy	Pancreatic sphincterotomy	*p* value
Performing center, *n* (%)				
Q1–3	256	236 (92)	20 (8)	0.249
Q4	120	106 (88)	14 (12)	
Performing endoscopist, *n* (%)				
Q1–3	276	258 (93)	18 (7)	0.007
Q4	100	84 (84)	16 (16)	

	Pancreatic sphincterotomy	Fatal outcome	Non-fatal outcome	*p* value
Performing center, *n* (%)				
Q1–3	Total	59 (23)	197 (77)	0.024
	Yes	9 (45)	11 (55)	
	No	50 (21)	186 (79)	
Q4	Total	16 (13)	104 (87)	1.000
	Yes	2 (14)	12 (86)	
	No	14 (13)	92 (87)	
Performing endoscopist, *n* (%)				
Q1–3	Total	62 (22)	214 (78)	0.138
	Yes	7 (39)	11 (61)	
	No	55 (21)	203 (79)	
Q4	Total	13 (13)	87 (87)	0.215
	Yes	4 (25)	12 (75)	
	No	9 (11)	75 (89)	

*Q* quartile

When a perforation occurred, it was more likely to get a fatal outcome both at a Q1–3 center and when a Q1–3 endoscopist performed the ERCP according to the univariate logistic regression results (Table 4).

There were no significant differences between the 62 patients with confirmed perforations and the 13 patients with missing or incorrect data (Table 6).

**Table 6:** Validation of the reviewed patients

	Reviewed patients	Not reviewed patients	*p* value
	*n* = 62	*n* = 13	
Age, median (Q1; Q3), years	78 (72; 84)	71 (64; 77)	0.118
Sex, *n*			0.171
Male	32	4	
Female	30	9	
ERCP indication, *n*			0.748
Malignancy diagnosis	45	10	
Papilla within diverticula, *n*	8	0	0.093
Bile duct stenosis, *n*	30	7	0.155
Sphincterotomy, *n*			
Bile duct	26	5	1.000
Pre-cut	16	5	0.497
Pancreatic duct	10	1	0.677
Bile duct stone extraction, *n*	5	0	0.580
Procedural time, median (Q1; Q3), min	40 (24; 65)	50 (45; 73)	0.471
Performing center, *n*			
Q1–3	49	10	1.000
Q4	13	3	
Performing endoscopist, *n*			
Q1–3	50	12	0.444
Q4	12	1	

*Q* quartile

There was a high rate of comorbidity in the 62 patients in the fatal group with reviewed patient records, 79% had malignancy and cardiovascular disease was present in 42%. In this group, only 23 of 62 procedures (37%) were successful. Stapfer II, III, and unclassified were the most frequent injuries. There were only two patients with a Stapfer I injury and none with Stapfer IV. Unclassified injuries were five esophageal and four gastric perforations, four stent perforations in the duodenum, and six perforations without known cause in the duodenum. Management after ERCP perforations in the fatal group is presented in Table 7 according to type of injury. No significant differences was seen between early, late, or no treatment according to the classification of injuries, between the different injury types. Post hoc tests were undertaken and showed no significant differences between treatment among Q1–3 and Q4 volume centers (*p* = 0.596) and endoscopists (*p* = 0.670) or if the patients had an age over 80 years or not (*p* = 0.329), neither if the patients had a malignancy or not (*p* = 0.921).

**Table 7:** Feature of injury and management of the 62 reviewed patients according to classification

Stapfer	Total	Management		
		Early treatment	Late treatment	Best supportive care/no treatment
I	2 (3)	2 (100)	0 (0)	0 (0)
II	21 (34)	6 (28)	10 (48)	5 (24)
III	20 (32)	10 (50)	4 (20)	6 (30)
IV	0 (0)	0 (0)	0 (0)	0 (0)
Unclassified	19 (31)	7 (37)	10 (53)	2 (10)

% in parentheses; *p* value 0.161

The median time from ERCP perforation until surgery or endoscopic management was 3 days (range 0–49) and time from ERCP perforation until death was 33 days (range 0–111).

The fatal group comprised nine deaths from multi-organ failure, six from shock, four from liver failure, three from cardiovascular failure, and a further three from respiratory failure. The remaining causes of death included malignancy: 44 of 62 patients (71.0%) had a malignancy. Eleven patients had potentially curable diseases.

## Discussion

This population-based, nationwide study concerning ERCP-related perforations identified malignancy, age over 80 years, and sphincterotomy in the pancreatic duct to be significant risk factors to die after a perforation.

The high incidence of sphincterotomy in the pancreatic duct among the fatal perforations is an important finding since pancreatic duct sphincterotomy has been launched as a valuable adjunct in difficult biliary cannulation [27], possibly being safer than pre-cut [28, 29]. However, extensive information on this subject in literature is lacking. The paucity of cases with perforation warrants additional large register studies to explore the risk of perforation after sphincterotomy of the pancreatic duct. The higher incidence of perforation associated with pancreatic duct sphincterotomy among Q4 endoscopists could be due to a selection of patients with pancreatic diseases, which are more challenging procedures, also requiring a more experienced endoscopist. Q4 endoscopists may be more persistent technically, possibly increasing the perforation risk. According to our results, these perforations were less likely to have a fatal outcome when performed at Q4 centers. This is possibly due to an earlier detection and/or the feasibility to cooperate with other highly specialized colleagues at Q4 centers, such as interventional radiologists.

In our study, the incidence of post-ERCP pancreatitis was high after a pancreatic duct sphincterotomy perforation but the pancreatitis had no impact on mortality. The connection between post-ERCP pancreatitis and sphincterotomy in the pancreatic duct has been corroborated in previous studies, which have also documented a reduced risk when using a pancreatic duct stent [27, 30].

Malignancy and age over 80 years were more frequent in the fatal perforation group than in the non-fatal group. This is in accordance with the previous studies showing increased post-ERCP mortality in older patients with comorbid conditions, e.g., cancer [10, 31, 32].

Sphincterotomy in the bile duct has been discussed and suggested as a risk factor for perforation [17, 18]. In this present study, bile duct sphincterotomy had a tendency to reduced mortality according to the univariate analysis. This could be explained by the lower incidence of bile duct stones in the fatal group. It has previously been shown that gallstone disease is associated with lower mortality after ERCP [10]. According to a large prospective study, this may be explained by a decreased risk of post-ERCP complications in this group [31]. Furthermore, therapeutic success in this setting is high by providing at least an adequate drainage [33].

An association between increasing endoscopist volume and decreasing adverse events has been reported [5]. This is despite the fact that the less-experienced practitioners probably rarely publish their data. Cotton et al. [18] concluded that pre-cutting is particularly hazardous for marginal indications and that ERCP including pre-cut should be done by endoscopists with adequate training and proven expertise, with standard techniques. Pre-cut has also been suggested as an risk factor for perforation [18], but a meta-analysis [34] did not notice a higher risk when pre-cut was used by experienced endoscopists. A prospective randomized multi-center study [35] demonstrated that, in expert hands, early pre-cut in difficult biliary cannulation is comparable to persistent standard cannulation, with respect to successful common bile duct access and overall complication rates, and is associated with a significantly lower risk of post-ERCP pancreatitis. In this current study, pre-cut was not a risk factor to die after a perforation.

Periampullary duodenal diverticula has been found to decrease the cannulation rate and to increase complications at ERCP [36]. However, as presented in a recent meta-analysis [37], duodenal diverticula has no impact on the perforation rate. This study showed no higher risk to die after a perforation with present periampullary diverticula.

The length of the procedure was no greater in the fatal group compared to the non-fatal group. Previously, Enns et al. [17] concluded that a longer duration of the procedure was a predictive risk factor for perforation.

Access to patient records in the non-fatal group would probably have strengthened the association between comorbidity and a fatal outcome. Among the patients with reviewed patient records (62), patients had multiple comorbidities, making them particularly susceptible to complications. This group also had a low ERCP success rate (37%). Conversely, completion of the intended ERCP procedure has been found to decrease mortality [15].

Management differs depending on type of injury, and different classification schemes have been suggested for the retroperitoneal perforations following ERCP. Stapfer et al. [38] classified injuries into four types based on the mechanism, the anatomical location, and severity of the injury, and suggested management for each type. Type I injuries are large and usually discovered during the ERCP procedure. They require immediate surgery or endoscopic enclosure. Both types II and III may be managed non-surgically but require close surveillance. Howard et al. [39] suggested a similar scheme and recommended early endoscopic stenting and medical treatment with broad-spectrum antibiotics for both type II and type III injuries. Patients who failed to respond required operative exploration. Wu et al. [23] published two management algorithms, one for perforations recognized at ERCP and one for those not recognized at ERCP, the latter depending on the presence of abdominal pain and computed tomography scan findings.

In our study, ten duodenal perforations could not be graded according to Stapfer classifications. Similar limitation of classification was also documented by Dubecz et al. [12]. In a systematic review in 2017 [24] comprising 305 patients (16 fatal), there were 58% Stapfer type II perforations, 18% Stapfer type I, 13% Stapfer type III, and 11% Stapfer type IV perforations. In our study, the most frequent injuries were Stapfer type II (34%), followed by Stapfer type III (33%), and unclassified (31%). We found only two Stapfer type I perforations (fatal group) which may be explained by the early detection of this type of injury and prompt therapy reducing the risk of a fatal outcome. We encountered no Stapfer type IV, which is not a true perforation and obviously less severe. Early diagnosis and treatment of perforations is crucial; the mortality in the present population-based series of ERCP-related perforations was 20% which appears higher than that reported by others [19–22]. Stapfer type I perforations are often recognized during ERCP. The importance of early detection and the risk of delayed diagnosis and treatment have been emphasized especially in Stapfer type II perforations [23, 39]. Stapfer type II and III perforations may be more difficult to diagnose during the ERCP. In these perforations, patients often exhibit ambiguous symptoms and, when they are suspected, there should be a low threshold for computed tomography [17]. From our study, it is difficult to provide firm advice regarding the management of ERCP-related perforations. However, it is obvious that multimodal treatment with expertise in surgical, endoscopic, and interventional radiological procedures is required as provided at large volume center as university hospitals.

One strength of our study is its population-based character with a large number of ERCPs included through a validated national registry. Study limitations are the retrospective approach and the fact that a registry-based model introduces a risk of bias and under-reporting. Although patient files were retrieved, some data may be lacking. The study would have been strengthened if we had been permitted to retrieve also the patient records in the non-fatal group. However, there were no major differences between the excluded 13 patients and the 62 with complete information. Only 3/75 (4%) of the fatal patients were incorrectly registered.

In conclusion, this large, population-based study shows that malignancy, age over 80 years, and sphincterotomy in the pancreatic duct are independent risk factors to die after an ERCP-related perforation. Some of the patients with malignancy could possibly have undergone surgery with a curative intent if they had not got a perforation. The risk of a fatal outcome in perforations after pancreatic duct sphincterotomy was reduced when occurred at a Q4-center. Thus, at least in the case of a complicated perforation a transfer to a Q4-center may be considered.
